# TUMORAL TISSUE SPECIFIC PROMOTER HYPERMETHYLATION OF DISTINCT TUMOR SUPPRESSOR GENES IN A CASE WITH NONSMALL CELL LUNG CARCINOMA: A CASE REPORT

**DOI:** 10.4103/0970-2113.45279

**Published:** 2008

**Authors:** Sulhattin Arslan, Tamer Dogan, Binnur Koksal, Malik Ejder Yildirim, Cesur Gumus, Sahenda Elagoz, Ibrahim Akkurt, Oztürk Ozdemir

**Affiliations:** 1 Department of Thorax Diseases, Faculty of Medicine, Cumhuriyet University 58140 Sivas/Turkey; 2 Department of Medical Genetics, Faculty of Medicine, Cumhuriyet University 58140 Sivas/Turkey; 3 Radiology, Faculty of Medicine, Cumhuriyet University 58140 Sivas/Turkey; 4 Pathology, Faculty of Medicine, Cumhuriyet University 58140 Sivas/Turkey

**Keywords:** Non-small cell lung carcinoma, Tumor suppressor genes, Epigenetic alterations

## Abstract

**Objective:**

Non-small cell lung carcinoma is an aggressive phenomenon and the epigenetical alterations of some tumor supressor genes have been reported for the different tumor types.

**Case Presentation:**

It is presented a case report concerning a 43 years old male with NSCLC on the lower segment of the right lung. The patient underwent a diag-nostic excisional thin-needle biopsy and after the histological confirmation. We examined the promoter methylation status of some distinct tumor supressor genes in tumoral and blood tissues of the case after sodium bisulfite conversion and DNA amplification with methylation specific multiplex PCR technique. Both tissues were also searched for G to A transitions in codons 12 and 13 of the K-ras proto-oncogene.

**Results:**

Tumor specimen showed fully methyl pattern profiles for the SFRP2, p16, DAPK1 and partially hyper-methylated profile for the p53 and MGMT genes in this case with non-small lung carci-noma. Blood speicemen showed normal hypomethylated profiles for all studied TS genes. The K-ras proto-oncogene was in normal structure both in blood and tumoral spiecemens that examined.

**Conclusion:**

Results indicate that genes exhibit tumor suppressor activi-ties in blood, but exhibit epigenetic inactivation in carcinoma cell. These findings strongly support the hypothesis that epigenetic mechanisms may play an important role in the non-small cell lung carcinogenesis in human.

## INTRODUCTION

The underlying basis of cancer is a cumulative series of genetic and epigenetic alterations leading to deregulated cell growth^1^. The genetic model of cancer appears to explain most rare cancer family synromes. It also appears correct for cancer initiation, i.e., for most common tumors mutations have been identified as early events^2^. Several oncogenes have been identified in lung cancer. Dominant oncogenes of the c-myc family are frequently overexpressed in both small (SCLC) and non-small cell lung cancer (NSCLC), while the K-ras oncogene is never mutated in SCLC but it is in 30% of NSCLCs^3^. Point mutations of the oncogene K-ras is found in 15 to 30% of adenoma carcinomas, especially in smokers^4^.

In the last decade, aberrations in DNA methylation patterns have been accepted to be a common feature of human cancer^5^. These aberrations includes hypomethylation leading to oncogene activation and chromosomal instability, hypermethylation and tumor suppressor gene silencing, and chromatin modification acting directly, and cooperatively with methilation chances, to modify gene expression^2^. Not every gene is methylated in every tumor type, but a strong specivity is apparent^6^. SFRP2 gene is claimed as a tumor suppressor gene that inactivated by epicenetic CpG hypermethylation especially in colorectal carcinoma^7^. However, SFRP2 promoter hypermethylation has been reported for other cancer types such as breast cancer^8^, gastric cancer^9^, hepatocellular carcinoma^10^ and renal cell carcinoma^11^. P16, the cell cycle inhibitor, is hypermethylated in a variety of human tumors and cell lines aloowing the cancer cell to escape senescence and start proliferating^12^. The promoter hypermethylation of MGMT, that prevents the removal of groups at the O^6^ position of guanine, leads to appearance of K-ras and P53 mutations^13^. The tumour suppressor hypermethylated in cancer 1 (HIC1) is a transcriptional repressor, which is epigenetically silenced in solid cancers^14^. Death-associated protein kinase (DAPK) is a calmoduline-regulated serine/threonine kinase and possesses apoptotic and tumor-suppressive functions^15^.DAPK 1 is an important tumor suppressor gene for variety of human cancer types including lung cancer^16^.

Lung cancer is the phenotypic concequence of an accumulation of genetic changes in airway epithelial cells that result in unrestrained celluler proliferation. In the current study it was aimed to compare the epigenetic modification of some distinct TS gene promoters in blood and tumor specimen in a case with non-small cell lung carcinoma.

## CASE

A 43-years old male patient was admitted for irritant cough and expectoration of bloody mucus in the previous two months. He had a family history of lung malignancy and alcohol and cigarette, but his dietary habits were normal. He has worked as a central heating system worker during last 22 years. His 48 years old brother was also operated due to the non-small cell lung carcinoma in 2005. The physical examinations, routine blood and urine tests of patient were normal.

### Patient and biological specimens

Tissue collection and analysis in this study were approved by the Research Ethics Committees of Faculty of Medicine of Cumhuriyet University. Pheripheral blood sample and tumoral tissue specimen were used for total genomic DNA isolation and epigenetic analysis. Tumor tissue chosen for analysis was routinely processed and microscopically examined for the regions enriched in neoplastic cells. For the correlation study of methyl patterns, two different tissues were analysed histopathological, immunohistochemical and epigenetically. The lysate was aliquoted into different tubes, each containing 20 µl of tissue lysate, and stored at -20°C until sodium bisulfite modification was performed. Direct in vitro amplification of the protooncogene K-ras and tumor supressor genes of SFRP2, p16, DAPK1, p53 and MGMT were performed by multiplex PCR based on epigenetic modification analysis.

### Analysis of mutation status of the K-ras oncogene

Sections (10 µm thick) of the fresh tumor specime and 100µl pheripheric blood samples with EDTA were used for genomic DNA isolation and in vitro gene amplification. Total genomic DNA was isolated by the nucleospin kit extraction technique (Invitrogene, Germany) with some modifications^17^. In vitro amplification of DNA fragments encompassing codons 12 and 13 of the K-ras oncogene was performed from blood and tumoral tissue biopsy. Twelve common muation regions of K-ras oncogene were simultaneously in vitro amplified and biotin-labelled in a single < multiplex > amplification reaction. PCR was performed in a Perkin Elmer 9600 and the profile consisted of an initial melting step of 2 minutes at 94°C; followed by 35 cycles of 30 seconds at 94°C, 30 seconds at 61°C, and 30 seconds at 72°C; and a final elongation step of 7 minutes at 72°C. The mutation analysis was performed by StripAssay technique (Vienna Lab, StripAssay GmbH, Austria) which is based on the reverse-hybridization principle automatically.

### Analysis of methylation status of the promoter region of tumor supressor genes

DNA methylation patterns in the promoter CpG islands were determined in both blood and tumoral tissue samples by methylation-specific PCR (MSP) following the bisulfite modification of isolated genomic DNA. Fortyfive µl of isolated DNA were denatured by alkalizer (final 0.3 mmol/L) at 55°C for 10 min and modified by sodium bisulfite (5.20-5.69 mol/L, pH 5.0, Viennalab, Austria) for 4 hr at 55°C in the dark. After incubation, bindig buffer was added (300 µl per sample) and lysate was transferred into receiver tube with spin filter, centrifuged at 13 000 rpm for 30 second. Filtrate was discarded, wash buffer (600 µl per sample) was added in spin filter and centrifuged at 13 000 rpm for 30 second. In the main time a mixture of alkalizer ethanol (1:10) was prepared. The mixture added into spin fitler 300 µl per sample, then incubated at room temperature for 30 minutes. After incubation, spin fitler was washed again with same procedure. Elution buffer (30 µl) was added into spin fitler, incubated 3 minutes at room temperature, centrifuged at 13000 rpm for 1 minute and spin fitler discarted. Resulting filtrate was keep at -20°C. Aliquots of 5 pL of bisulfite modified DNA were used for MSP reactions. Primers for a methylated and unmethylated promoter of the different TS genes were used and multiplex PCR based amplification procedure (Viennalab, Austria) was performed for the in vitro gene amplification. Amplification was carried out in a Bioer XP thermal cycler after 15 min at 94°C for Pre-PCR, 45 second at 94°C for denaturation, 45 second at 66°C for annealing and 45 second at 72°C for polymerisation of 45cycles of with final extension for 7 min at 72°C. Modified DNA from blood and tumoral tissue (10 pL of each PCR reaction) were compared in revers hybridisation stripAssay technique (ViennaLab, Austria).

CT scan of the chest showed an anomalous round solid tumor in the lower segment of the right lung (Figure 1A). The density of the mass was not uniform, and tumor was diagnosed as a primary lung cancer. Pathological examination of the tumor from the lung tissue revealed a non-small cell lung carcinoma after CT, histopathological and immunochemical examinations (Figure 1B,Figure 2). Pathological examination of the NSCLC tumor (5 cm in diameter) from the lung also revealed invading the pleura and lymph nodes (2 cm in diameter) but not the vessels. The current case and his operated brother were both heavy smokers and work in charcoal heating station for a long time. The case was still alive 05 mo after operation. Blood and thin-nedlee tumoral biopsy were taken for epigenetical analysis after fiber-optic bronchoscopy (FOB). Tumoral and blood tissues were analised for G to A transitions in codons 12 and 13 of the K-ras proto-oncogene and tissue specific epigenetic alterations. Tumor specimen was showed fully methyl pattern profiles for the SFRP2, p16, DAPK1 and partially hypermethylated profile for the p53 and MGMT genes in a case with non-small lung carcinoma (Figure 3,Table I). Blood speicemen showed normal hypomethylated profiles for all studied TS genes. The K-ras proto-oncogene was in normal structure both in blood and tumoral spiecemens that examined Figure 3.

**Fig 1A:**
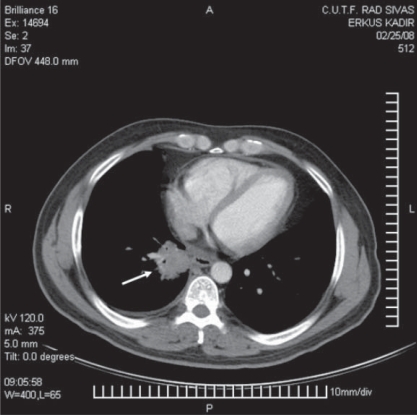
Panoramic radiograph of right lung of current case. Arrow indicates the enlargement tumor appearence

**Fig 1B:**
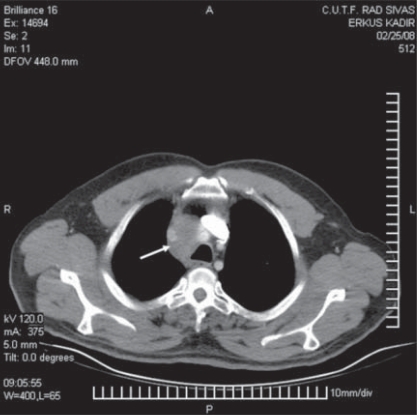
Panoramic radiograph of right lung shows (arrow) parathreacheal lymphadenopathy profile in the current case with non-small cell lung carcinoma

**Fig 2:**
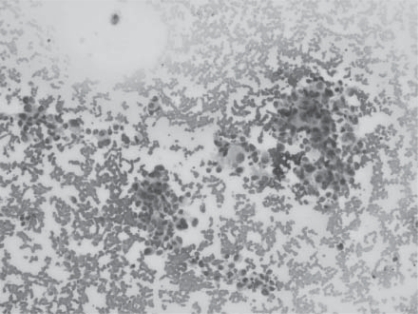
Hyperproliferative epithelial cell with vacoulised cytoplasm enlargement nuclei. (×20 ;MG)

**Fig 3:**
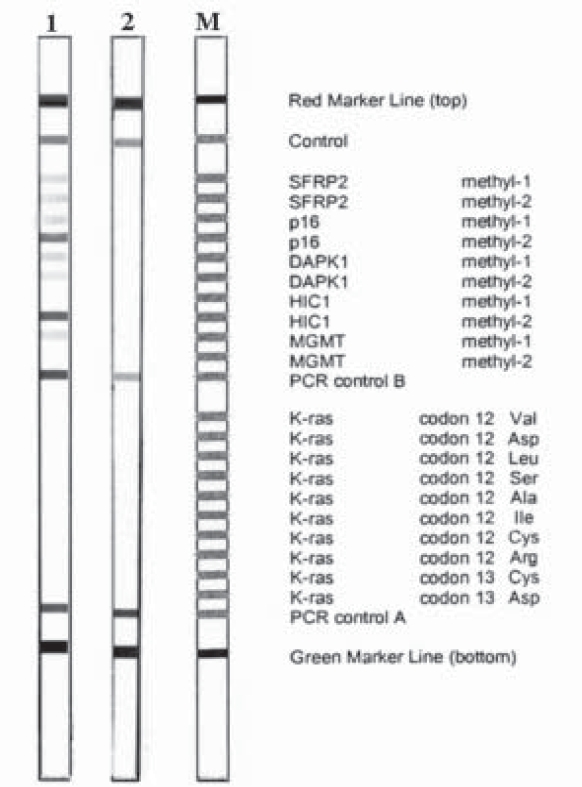
Shows epigenetically modified profiles of the TS genes of SFRP2, p16, DAPK1, p53, MGMT and point mutations in codon 12 and 13 of oncogene K-ras in blood and tumoral tissue samples of the current case.

**Table 1:** The tissue specific methylation profiles of promoter subunits of some distinct TS genes in blood and tumor samples from the current case with non-small cell lung carcinoma.

GENE TYPE	Blood	Tissue	Biopsy
SFRP2	Unmethylated, active gene		Fully methyleted, inactive gene
p16	Unmethylated, active gene		Fully methyleted, inactive gene
DAPK1	Unmethylated, active gene		Fully methyleted, inactive gene
p53	Unmethylated, active gene		Partially methyleted, heterozygous inactive gene
MGMT	Unmethylated, active gene		Partially methyleted, heterozygous inactive gene

## CONCLUSION

Lung tissue carcinomas especially recurrent NSCLC are most frequent reason of cancer related deaths all over the world^18^. We investigated the association between gene methylation and tumoral differentiations. We also showed different epigenetical patterns of distinct TS genes in two different tissues in one case with NSCLC. This status is due to epigenetic alterations like other cancer types as well as the more tendency of this type cancer to environmental conditions such as working conditions or smoking status. As indicated some researchers the genetic alterations contributing to cancer initiation, epigenetic alterations have important role in carcinogenesis, tumor invasion and metastasis. The presented case was heavy smoker, working in bad conditions and has a familial history of lung carcinoma. Tumoral specimen was showed fully methyl pattern profiles for the SFRP2, p16, DAPK1 and partially hypermethylated profile for the p53 and MGMT genes in the current case. Blood tissue has normal epigenetic profiles for all studied TS genes. No point mutation in K-ras proto-oncogene was detected in both blood and tumoral spiecemens that examined.

Our data also suggest that an important role of DAPK1 and other TS genes for fully and /or partially silencing through promoter CpG island hypermethylation in the development of NSCLC and the detection of aberrant hypermethylation on DAPK1 promoter from tumoral samples has potential clinical implications as a non-small lung tumor progression. Epigenetic modifications, such as DNA methylation, histone acetylation/deactylation, and histone methylation, are currently believed to play a major role in human cancer. The inactivation of the p16 gene in murine cancers induced by NNK most likely arises as a late event via homozygous deletion as claimed by Belinsky^19^. All of those changes of non-small cell lung cancer metastasis and occurred early in neoplastic evolution may be induced by DAPK1 and other tumor supressor genes. These findings verify that the DNA hypermethylation of some distinct tumor suppressor genes may play an important role for the initiation and/or progression of malignant lung carcinomas. Exposing to the chemicals such as smoke and or coak dust may initiate the tissue specific tumorogenesis. According to the above findings it is possible to discuss that different tumoral epigenetic profiles need alternative therapy for a good life quality and prognosis.

### Lanes

**Lane 1:** Gene profiles of the tumoral tissue shows epigenetically modified promoter region of the distinct tumor suppressor genes. Fully hypermethylated - inactive gene profiles of the TS tumor supressor SFRP2, p16, and DAPK1. The TS p53 and MGMT genes were showed partially hypermethylated and heterozygous inactive gene profiles in tumor cells. The K-ras proto-oncogene was in normal structure.

**Lane 2:** The gene profiles of the blood tissue shows fully hypomethylated -active gene profiles of the TS tumor suppressor genes in blood cells in the current case. The K-ras proto-oncogene was in normal structure as tumor cells.

**M** - Standart size marker(Vienna Lab, StripAssay)
